# Editorial: Improving the nutritional content and quality of plants: promises, achievements, and future challenges, volume II

**DOI:** 10.3389/fpls.2023.1253581

**Published:** 2023-07-28

**Authors:** Felipe Klein Ricachenevsky, Marta Wilton Vasconcelos, Huixia Shou, Alexander Arthur Theodore Johnson, Raul Antonio Sperotto

**Affiliations:** ^1^ Botany Department, Institute of Biosciences, Graduate Program in Cellular and Molecular Biology, Center for Biotechnology, Graduate Program in Botany, Federal University of Rio Grande do Sul, Porto Alegre, RS, Brazil; ^2^ Universidade Católica Portuguesa, CBQF - Centro de Biotecnologia e Química Fina - Laboratório Associado, Escola Superior de Biotecnologia, Porto, Portugal; ^3^ The State Key Lab of Plant Physiology and Biochemistry, College of Life Sciences, Zhejiang University, Hangzhou, China; ^4^ School of BioSciences, The University of Melbourne, Melbourne, VIC, Australia; ^5^ Graduate Program in Biotechnology, University of Taquari Valley - Univates, Lajeado, Brazil; ^6^ Graduate Program in Plant Physiology, Federal University of Pelotas, Pelotas, Brazil

**Keywords:** nutritional quality, nutrients, food safety, biofortification, food security

Human nutrition is based to a large extent on the amount of nutrients found in seeds, leaves and roots of edible plants. Increasing the nutritional quality of foods – biofortification – has been a primary goal of research in the field of plant nutrition to overcome hidden hunger – the lack of proper nutrients in the diet. Biofortified crops also need to be as productive as regular genotypes and avoid hazardous elements, which often increase concentration when desired beneficial ones accumulate. In a scenario of climate change, which may lead to even lower micronutrient concentrations in seeds, combined with the need to make crops more resilient to environmental extreme events (Eckardt et al., 2023), clearly calls for understanding plants genetics, biochemistry and physiology to achieve the goals of biofortification, as well as translating them into action. In this second volume of our Research Topic, are described different approaches to improving nutritional quality of plants.

Delivering biofortified crops is not an easy task, since it involves not only the science of generating nutrient-dense varieties but also getting them to the people that need the most. Biofortification crops that are locally relevant involves several steps, including crop selection, identifying diversity for the trait, evaluation of farmers and consumers acceptance, selection of high yield genotypes for breeding, crop implementation and deregulation, monitoring, among others. Birol and Bouis review the importance of such socio-economic, multidisciplinary research in the context of HarvestPlus, the major program that has been delivering several biofortified crops around the world through breeding. The case of HarvestPlus is exemplary to other biofortification efforts, and this review calls attention to the need for careful considerations of the larger context in which nutrient-dense crops will be used.

Biofortification efforts depend largely on basic science to be feasible. Despite the knowledge accumulated in the model species *Arabidopsis thaliana* and rice, wheat, despite being one of the world’s most relevant cereal, is relatively less investigated regarding nutrient homeostasis regulation. Kumar et al. reviews iron (Fe) homeostasis with a focus on biofortification. The authors identify wheat likely orthologous genes for several known molecular players in Fe homeostasis in other plants; the approaches used to increase Fe concentration and bioavailability in wheat grains; and discuss bottlenecks in research that need to be overcome to increase Fe concentration. The work provides a nice summary and point to future directions for wheat biofortification by biotechnological and breeding approaches.

The rapid increase in the world’s population is causing food insecurity by less food availability and by the malnutrition of essential nutrients and vitamins. Biofortification strategies aiming to enhance the nutritional quality of edible crops have gained popularity to improve micronutrient contents. Inoculation of *plant growth-promoting rhizobacteria* (PGPR) can enhance the uptake of micronutrients (N, P, Fe, Zn, and Cu) and promote mineral biofortification in cereal grains, through enhanced nutrient solubilization and siderophores/exopolysaccharides production. Ahmad et al. investigated the biofortification potential of pre-isolated and characterized Zn solubilizing PGPR strains in maize plants. According to the authors, microbial inoculants played a significant role in enhancing the growth, yield, and quality of maize, and could be used as a biofertilizer for Fe and Zn biofortification and sustainable production.

Worried with the serious impacts that climate change together with the pandemic outbreak can have in agriculture production, socioeconomic insecurities, and health implications globally, Babele et al. highlight the importance of mainstreaming climate-resilient and low input crops with more contemporary agriculture practices. According to the authors, one possible solution to tackle these issues would be the cultivation of underutilized/neglected crops to diversify crop production and provide more nutritious food sources. Of the orphan crops, millets are the world’s ancient and most versatile grains, playing a vital role in food security and human nutrition/health. Therefore, this review focus on the major barriers for millets improvement, pre- and post-harvest technologies, and policies required to introduce and establish millets in mainstream agriculture.

Besides nutrient concentration, consumer-relevant traits such as appearance and cooking characteristics, including overall taste, should be considered. In rice, the Wx locus controls amylose content, which affects rice grain quality. Xia et al. evaluated different alleles of *Wx* genes in near isogenic or transgenic lines under varying nitrogen fertilization and high temperature stress. The authors conclude that specific alleles that confer medium amylose levels, combined with low nitrogen fertilization confer better grain quality, whereas high temperature decreases quality. In a climate change scenario, where high temperatures may become more frequent in rice farming areas, the work is important for considering how agronomic practices and the environment affect final product appearance and cooking traits.

Another important plant species that is becoming more popular is cashew (*Anacardium occidentale* L.). The edible parts are the nut (true fruit) and the apple (false fruit). Dakuyo et al. have evaluated 30 cashew accessions from Burkina Faso, for morphophysiological characteristics, agronomic traits, nutrient and anti-nutrient concentrations. The nut is rich in protein and fat, and the apple in carbohydrates, and they vary in their elemental profiles, as well as in phytate, showing potential for selection. The work could be an important starting point for further development of nutrient-enriched cashew.

Grasslands are important for livestock grazing, but areas have been suffering pressure from agriculture and cattle number expansion. Grassland cultivation is an important alternative for sustainable agriculture, but best practices need to be used to provide quality forage for animals. Li et al. tested growing oat (*Avena sativa*), common vetch (*Vicia sativa*), or both species simultaneously, and simulated either grazing or hay production. Overall, mixed sowing and simulated grazing increased forage yield, but crude protein and fat decreased with yield. Authors conclude that sowing both species and simulated grazing was the best combination of methods for better grassland use.

As it’s becoming increasingly clear, feeding the predicted nine billion people by mid-century will be challenging. Hidden hunger, a problem linked to the adoption of cereal-centered diets, has to be considered in strategies to increase agriculture productivity. The papers in this second volume of our Research Topic further advance diverse approaches to increase nutritional quality in plants, and can be important for pointing new, innovative directions to achieve a sustainable food production system.

## Author contributions

All authors listed have made a substantial, direct, and intellectual contribution to the work and approved it for publication.
